# FindMyApps eHealth intervention improves quality, not quantity, of home tablet use by people with dementia

**DOI:** 10.3389/fmed.2023.1152077

**Published:** 2023-05-30

**Authors:** David P. Neal, Leanne Kuiper, Daniela Pistone, Channah Osinga, Sanne Nijland, Teake Ettema, Karin Dijkstra, Majon Muller, Rose-Marie Dröes

**Affiliations:** ^1^Department of Psychiatry, Amsterdam University Medical Centres Location Vrije Universiteit, Amsterdam, Netherlands; ^2^Mental Health Program, Amsterdam Public Health Research Institute, Amsterdam, Netherlands; ^3^Institute for Interdisciplinary Studies, University of Amsterdam, Amsterdam, Netherlands; ^4^Faculty of Social and Behavioral Sciences, Universiteit Utrecht, Utrecht, Netherlands; ^5^Faculty of Behavioral and Movement Sciences, Vrije Universiteit Amsterdam, Amsterdam, Netherlands; ^6^School of Health, Saxion University of Applied Sciences, Deventer, Netherlands; ^7^Department of Internal Medicine Section Geriatrics, Amsterdam University Medical Centers, Amsterdam, Netherlands; ^8^Amsterdam Cardiovascular Sciences Research Institute, Amsterdam, Netherlands

**Keywords:** dementia, eHealth, social health, clinical trial, process evaluation

## Abstract

**Introduction:**

FindMyApps is a tablet-based eHealth intervention, designed to improve social health in people with mild dementia or mild cognitive impairment.

**Methods:**

FindMyApps has been subject to a randomized controlled trial (RCT), Netherlands Trial Register NL8157. Following UK Medical Research Council guidance, a mixed methods process evaluation was conducted. The goal was to investigate the quantity and quality of tablet use during the RCT, and which context, implementation, and mechanisms of impact (usability, learnability and adoption) factors might have influenced this. For the RCT, 150 community dwelling people with dementia and their caregivers were recruited in the Netherlands. For the process evaluation, tablet-use data were collected by proxy-report instrument from all participants' caregivers, FindMyApps app-use data were registered using analytics software among all experimental arm participants, and semi-structured interviews (SSIs) were conducted with a purposively selected sample of participant-caregiver dyads. Quantitative data were summarized and between group differences were analyzed, and qualitative data underwent thematic analysis.

**Results:**

There was a trend for experimental arm participants to download more apps, but there were no statistically significant differences between experimental and control arm participants regarding quantity of tablet use. Qualitative data revealed that experimental arm participants experienced the intervention as easier to use and learn, and more useful and fun than control arm participants. Adoption of tablet app use was lower than anticipated in both arms.

**Conclusions:**

A number of context, implementation and mechanism of impact factors were identified, which might explain these results and may inform interpretation of the pending RCT main effect results. FindMyApps seems to have had more impact on the quality than quantity of home tablet use.

## 1. Introduction

Dementia, or major neurocognitive disorder, is a growing public health problem. By 2050, over 130 million people worldwide are expected to be living with dementia, which remains incurable (1, 2). Family and informal caregivers of people with dementia may also experience adverse health outcomes, and this may have been exacerbated by the recent COVID-19 pandemic (3, 4). Cost-effective solutions are required to support people with dementia and their caregivers to live independently and maintain quality of life (5). Good quality of life in dementia depends not only on good physical and mental health but also on good social health (6, 7). Social health in dementia comprises meeting one's potential and obligations, self-management and social participation (8). To support social health, digital tools provide an opportunity for scalable, yet personalized, solutions (2, 9–11). Unfortunately, few high-quality studies have evaluated the effectiveness of digital interventions for social health in dementia (12–14).

FindMyApps is a tablet-based intervention designed to improve social health in dementia, and is currently the subject of a large-scale randomized controlled trial (RCT) (15–18). FindMyApps aims to help people find, install and learn to use tablet apps which meet their personal needs and interests. FindMyApps comprises: a tablet computer (running the iPadOS or Android operating system) with the FindMyApps app (a personalized app-selection tool, linked to a database of apps assessed as generally user-friendly in dementia and organized into categories by topic); and training (including a written manual) covering the use of the tablet and the FindMyApps app and, for caregivers, how to support people with dementia to learn to use the tablet. FindMyApps is expected to provide users with the capability and motivation to use the tablet, and the opportunity to find relevant and user-friendly apps for self-management and social participation, and would therefore be expected to result in particular behavioral outputs (19). It is anticipated that participants will, as advised: engage with training sessions by video call; make use of available learning resources (handbook, instruction films and telephone helpdesk); practice with the tablet for at least eight 30-min sessions; and search for and download apps, if necessary with help from their caregiver. People should accordingly learn to use the tablet with increasing independence, adopting the use of tablet-based apps which can improve their self-management and social participation.

As a complex intervention, FindMyApps has been developed and evaluated following the relevant UK Medical Research Council (MRC) framework, which includes the recommendation to conduct a process evaluation alongside main effect studies, “…to explore how and under what circumstances outcomes are achieved” (20–22). Factors which should be investigated relate to context (personal, environmental and social factors), implementation (what was delivered to trial participants by investigators) and mechanisms of impact (how participants interacted with the intervention). Insights from participants with respect to these factors are essential to the development and evaluation of person-centered care. However, achieving accurate recall and avoiding overburdening participants is challenging in the context of dementia (23). Caregiver proxy-reports are also subjective, and therefore susceptible to systematic effects such as social desirability bias (24, 25). Proxy and self-reports may also differ considerably, complicating interpretation (26). An objective measure of tablet and app use may aid interpretation of results, though such data cannot capture the full experience of participants (24). In this paper we therefore report results from a mixed methods process evaluation, conducted alongside a large-scale RCT investigating the effectiveness of FindMyApps in improving social participation and self-management of people with mild dementia. The goal is to investigate the extent to which the anticipated behavioral outputs of the intervention and therefore the RCT outcomes, may have been influenced by context, implementation, and mechanism of impact factors. The results of this process evaluation will inform the interpretation of RCT outcomes, suggest hypotheses for *post-hoc* analyses on outcome data, inform future implementation of FindMyApps, and inform evaluation of other digital interventions.

## 2. Methods

### 2.1. Design

This process evaluation was nested in a non-blinded, randomized controlled trial, comparing the effect of the FindMyApps intervention to a control intervention (digital care as usual) on social participation and self-management of people with dementia (or mild cognitive impairment, MCI) after 3 months. A detailed description of the RCT protocol has been published elsewhere (18). In this parallel mixed methods design, quantitative and qualitative data regarding context, implementation and mechanisms of impact factors were collected simultaneously, alongside RCT outcome data (Table 1 provides an overview of data collected for the process evaluation). “Mechanisms of impact” was conceptualized as comprising usability, usefulness, learnability and adoption. Usability was further divided into perceived effectiveness, efficiency and user satisfaction, based on International Organization for Standardization (ISO) definitions, which consider the quality of a digital tool when used by a particular user, for a particular goal, under particular conditions (27, 28). The GRAMMS checklist for mixed methods research and “consolidated criteria for reporting qualitative research” (COREQ) framework were followed in planning, executing and reporting the research (29, 30).

**Table 1:** Data collected to investigate context, implementation and mechanism of impact factors as part of the process evaluation.

**Factor groups**	**Data collected**	**Method**	**Timepoints**	**Participants (*n*)**
Context	Personal, social & financial factors perceived to have influenced outcomes	CGPRI	T0, T1, T2, T3	All caregivers (150)
		SSI-P	T3	Purposively-selected dyads (30)
		SSI-I	After data collection	Investigators (3)
	Participants' experiences regarding research method	SSI-P	T3	Purposively-selected dyads (30)
		SSI-I	After data collection	Investigators (3)
Implementation	Reported tablet use frequency	CGPRI	T0, T1, T2, T3	All caregivers (150)
	Reported number of apps downloaded per participant	CGPRI	T0, T1, T2, T3	All caregivers (150)
	Reported number of apps used per participant	CGPRI	T0, T1, T2, T3	All caregivers (150)
	Observed frequency & duration of use of FMA	AAUD	Continuous (T0–T3)	Experimental arm participants (75)
	Observed number of apps on which “download” clicked per participant	AAUD	Continuous (T0–T3)	Experimental arm participants (75)
	Perceived functioning of tablet & FMA	SSI-P	T3	Purposively-selected dyads (30)
		SSI-I	After data collection	Investigators (3)
	Perceived appropriateness & success of online training	SSI-P	T3	Purposively-selected dyads (30)
		SSI-I	After data collection	Investigators (3)
	Users' experiences of learning to use the tablet & FMA	SSI-P	T3	Purposively-selected dyads (30)
**Mechanism of impact**				
Usability	Observed frequency with which categories of apps from FMA were viewed or downloaded	AAUD	Continuous (T0–T3)	Experimental arm participants (75)
	Participants perceptions of effectiveness, efficiency & satisfaction with interventions	SSI-P	T3	Purposively-selected dyads (30)
Usefulness	Perceived usefulness of the tablet & FMA	SSI-P	T3	Purposively-selected dyads (30)
Learnability	Observed frequency of use of training features in FMA	AAUD	Continuous (T0–T3)	Experimental arm participants (75)
	Reported nature & frequency of use of FMA helpdesk	SSI-P	T3	Purposively-selected dyads (30)
	Reported nature & frequency of use of FMA helpdesk	SSI-I	After data collection	Investigators (3)
	Participants' experiences of learning to use tablet & FMA	SSI-P	T3	Purposively-selected dyads (30)
Adoption	Reported frequency of tablet & FMA use at final follow-up and intention to continue using	SSI-P	T3	Purposively-selected dyads (30)

FMA, FindMyApps app; CGPRI, caregiver proxy-report instrument; SSI-P, semi-structured interview with trial participants; SSI-I, semi-structured interview with investigators; AAUD, automatically collected app-usage data; T0–3, 0, 1, 2 or 3 months, respectively.

### 2.2. Ethics and trial registration

This study was approved by the Medical Ethics Committee of the VU medical center (2019.605) and the Scientific Quality Committee of Amsterdam Public Health Research Institute (SQC 2019–065). The trial is registered in the Dutch Trial Register (NTR NL8157).

### 2.3. Participants

Participant dyads (people with dementia and caregivers) were recruited from the RCT sample, and investigators providing the interventions. A target of 150 dyads to be included in the RCT was based on a power calculation performed using G^*^Power version 3.1 for main effects MANOVA on primary RCT outcomes, for two dependent variables, two groups, alpha = 0.05 and power = 0.8, and a moderate effect size (eta-squared = 0.06). All participants provided written and verbal informed consent to participate. All caregivers enrolled in the RCT (experimental and control arms; *n* = 150) were asked to complete proxy report instruments. FindMyApps app usage data were collected automatically from dyads in the experimental arm (*n* = 76). Between February 2020 and September 2021, 30 RCT dyads (15 experimental arm, 15 control arm) were purposively selected (sampling participants to achieve a range of in age, tablet experience, and relationship between the caregiver and person with dementia) to participate in a one-off semi-structured interview (SSI). This sample (10% of RCT participants) was the largest feasible number of SSIs within the confines of the resources available for the study. In March 2022, investigators who had administered the FindMyApps training were also asked to participate in SSIs.

### 2.4. Intervention

The FindMyApps and control interventions were implemented as described in the study protocol (18). In the experimental arm, participants received all components of the FindMyApps intervention, including advice to practice at least two times a week, and the option to call a helpdesk with questions or problems. In the control arm, participants also received training in the use of the tablet and downloading apps, which included provision of a handbook (including links to websites with lists of apps recommended for people with dementia), a training film and the option to call the helpdesk with questions or problems. In both arms, the training sessions were provided by trained investigators by video-call because of restrictions associated with the COVID-19 pandemic. The training manuals were digitally provided to participants before the video-call.

### 2.5. Data collection methods and procedures

An overview of the data collected for this process evaluation is shown in Table 1.

#### 2.5.1. Caregiver proxy-report instrument

At baseline (T0) and after one, two- and 3-months intervention (T1, T2, T3), caregivers completed a telephone-administered questionnaire about tablet-use over the preceding 4 weeks. They indicated how often their partner had used the tablet, with answers scored on a scale from 1 (less than once a week) to 4 (every day), listed any apps which they or their partner had downloaded or used, and reported on their experiences and any “significant events” which had influenced their tablet use. Investigators entered data directly into the electronic database Castor EDC.

#### 2.5.2. Automatically-collected app-usage data

The software Matomo 3.6 automatically registered data pertaining to use of the FindMyApps app. All activity was associated with a unique “user id” variable, which represented a single user across multiple sessions. The Matomo user id was separate from the participant's trial identification number. Only the investigators held the key relating the Matomo user id to the trial identification number. Page views, events and actions were timestamped and associated with a unique “visit id” variable, representing a single continuous session using the app. The duration of each session was recorded. IP addresses of participants were masked and no other personal identifying information was registered. Information of interest was computed from raw data variables: per user—the number of FindMyApps use sessions, duration of FindMyApps sessions, number of apps on which “download” was clicked, and number of clicks on the “Training” button to view training films; and per category (topic) of app—the number of page views and number of clicks made to download apps from that category.

#### 2.5.3. SSIs with trial participants and investigators

Interview guides (see Supplementary material) were devised by DN and RMD, adapted from a guide used in a pilot trial of FindMyApps (31). Questions specifically targeted the factors relevant to this process evaluation, in the context of each trial arm. The interview guide for investigators covered similar content to the interviews with RCT participants. All interview guides included open questions, closed questions (responses selected from ordinal or Likert scales or binary yes/no options), and hybrid questions which asked for a categorical response and provided the option to further elaborate. Trial participants knew the interviewers and were aware of their occupations, research focus and motivations. They were interviewed in their own home, by telephone, with no-one else present and interviews were expected to last 30–40 min. Interviews were conducted by two interviewers, DN (male, physician) and either LK, DP, CO, or SN (female, master students cognitive sciences or neuropsychology). Interviews with the investigators were carried out face-to-face at their office. Interviewers were trained in good clinical practice, and communication with people with dementia. In each case, one interviewer transcribed the conversation and made contemporaneous field notes. Transcripts were not returned to those interviewed.

### 2.6. Data analysis

Data regarding background characteristics of RCT participants were described and analyzed using SPSS v28. Differences between experimental and control arms were tested, depending on level of measurement, with the Student's *t*-test, Mann-Whitney *U*-test, or Pearson Chi-squared tests.

Data from the caregiver proxy-report instrument and the automatically-collected FindMyApps app-usage data were cleaned and analyzed using the software R v4.2.1. Analyses were performed using data from those who completed the study, including follow-up. Reported frequency of tablet-use was scored at each time-point and summed per participant (generating a scale with range 3–12). The lists of apps downloaded and used at T1, T2, and T3 were used to estimate the total number of apps downloaded and used per dyad during the study. Mann-Whitney *U*-tests were carried out to investigate between-group differences. Confounders and predictors of outcomes were further investigated with binomial logistic regression. Automatically collected app usage data regarding duration and number of sessions, and download attempts were visualized at user-level, and page views and download attempts at the app category-level. Correlations between observed measures of activity per user, and between observed and reported number of apps downloaded were investigated.

Responses to SSI closed questions were summarized (percentage agreement with response options or mean and standard deviation, as appropriate). Owing to the small sample sizes and the fact that many interview questions differed between the experimental and control arms, no between-arms statistical difference tests were performed. Analysis of responses to open questions was grounded in a contextualist epistemological approach, employing codebook thematic analysis (32, 33): an initial coding framework was based on the MRC process evaluation guidance and the intervention logic model. Following initial familiarization with the data, the coding framework was refined and all transcripts were coded by two investigators (junior/senior) working independently (DN, LK, DP, CO, SN, TE, RMD). Inter-rater reliability was not measured but coding discrepancies were discussed with a senior researcher (RMD) and codes adjusted when consensus was reached. The software MaxQDA 2020 was used to manage and analyze data. Coding frequencies, polarity (negative, neutral, or positive with respect to the topic discussed), and important points with respect to each MRC factor were summarized, with illustrative quotations translated from Dutch by a bilingual investigator (DN). A lay summary of key findings was shared by email with RCT participants, but no feedback was received.

Results were presented with respect to the three MRC factors (context, implementation and mechanisms of impact). Quantitative and qualitative data were integrated by DN in a triangulation matrix, to identify (partial) agreement or disagreement (29, 34, 35). All types of data were given equal weight.

## 3. Results

### 3.1. Characteristics of trial participants

**Figure 1:**
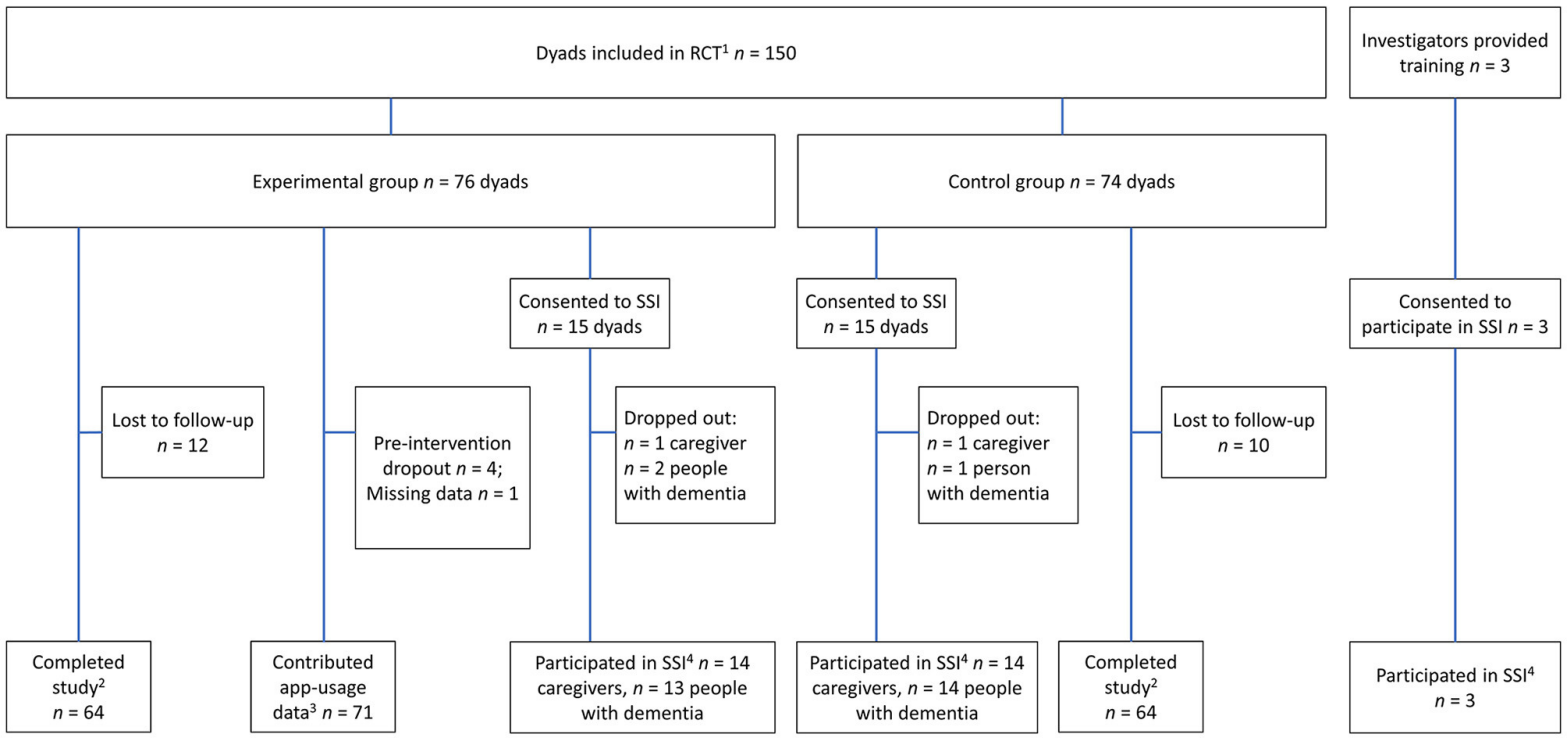
RCT participants and investigators from whom data was collected for use in this process evaluation. 1—Provided demographic data. 2—Provided full set of proxy report data (CGPRI, see Table 1). 3—Provided automatically collected app-usage data (AAUD, see Table 1). 4—Provided qualitative data in the semi-structured interview (SSI-P and SSI-I, see Table 1).

Figure 1 illustrates how participants were sampled and which data were collected. Background characteristics of all RCT participants, and the subgroup who took part in SSIs are presented in Table 2. Background characteristics did not differ significantly between trial arms.

**Table 2:** Background demographic characteristics of trial participants.

	**All trial participants**				**Trial participants interviewed in SSIs**			
	**Experimental (*****n*** = **76)**	**Control (*****n*** = **74)**	**Difference test**	***p***	**Experimental (*****n*** = **15)**	**Control (*****n*** = **15)**	**Difference test**	***p***
Mean age PwD (SD) [range]	73.2 (9.5) [50–95]	72.4 (8.8) [53–93]	*t* =0.522	0.603	70.6 (10.9) [50–88]	72.4 (11.6) [54–90]	*t* = 0.437	0.665
Gender PwD Female, *n (%)*	34 (44.7)	29 (39.2)	χ2 = 0.474	0.491	9 (60.0)	8 (53.3)	χ2 = 0.136	0.713
Diagnosis PwD, *n*			χ2 = 0.002	0.968			χ2 = 2.222	0.136
MCI	29	28			4	8		
Dementia	47	46			11	7		
Mean BCRS PwD (SD) [range]	23.8 (3.8) [17–32]	23.8 (4.1) [17–33]	*t* = 0.075	0.940	22.7 (3.8) [17–30]	24.7 (4.6) [18–32]	*t* = 1.334	0.193
PwD had ever used a tablet before enrolment, *n* (%)	47 (61.8)	47 (63.5)	χ2 = 0.045	0.832	8 (53.3)	8 (53.3)	χ2 = 0.000	1.000
PwD highest education, *n*			χ2 = 3.395	0.183			χ2 = 2.489	0.288
Primary	18	17			1	1		
Secondary	15	24			7	3		
Tertiary	43	33			7	11		
Mean age CG (SD) [range]	65.4 (11.4) [25–87]	62.5 (14.3) [17–88]	U = 3,060.0	0.351	59.0 (16.6) [25–82]	58.1 (16.8) [17–79]	U = 113.5	0.967
Gender CG Female, *n (%)*	54 (71.1)	56 (75.7)	χ2 = 0.410	0.522	10 (66.7)	12 (80.0)	χ2 = 0.682	0.409
CG highest education, *n*			χ2 = 0.214	0.898			χ2 = 0.424	0.809
Primary	12	11			1	2		
Secondary	26	28			6	5		
Tertiary	38	35			8	8		
Relationship CG-PwD, *n*			χ2 = 0.009	1.000			χ2 = 1.529	0.676
Partner	57	55			9	10		
Son (in-law)/daughter (in-law)	13	13			4	3		
Sibling	1	1			0	1		
Other	5	5			2	1		

Pearson's chi-squared test was used to compare nominal variables between groups. Student's *t*-test was used for normally distributed continuous variables. Mann-Whitney *U*-test was used for non-normally distributed continuous variables. PwD, person with dementia; CG, Caregiver; MCI, Mild cognitive impairment; BCRS, Brief cognitive rating scale.

### 3.2. Coding scheme from thematic analysis of SSIs

After familiarization with the data, one revision was made to the coding framework: a branch was added to the context codes. The final list of codes is presented in Table 3, with coding frequency and polarity from the RCT participant SSIs, per trial arm. A trend for fewer negative comments and more positive comments from experimental arm participants is noted, particularly with respect to implementation and mechanisms of impact (particularly efficiency, user satisfaction and usefulness). Differences with respect to learnability were less marked, and comparatively more negative comments in the experimental arm appeared in relation to adoption. Regarding context, most comments in both groups relating to the research method were negative, and most comments regarding the intervention were neutral. Coding frequencies from the investigator SSIs were: context *n* = 27 (15 negative, seven neutral, five positive), implementation *n* = 77 (49 negative, three neutral, 25 positive), and mechanism of impact *n* = 13 (three negative, five neutral, five positive). Citations were assigned a code indicating whether the quotation was from a person with dementia or MCI (PWD), a caregiver (CG), or an investigator (INV).

**Table 3:** Frequency of coding by polarity of answers (negative, neutral, positive answers) to the open questions of the SSIs and trial arm.

	**Frequency of coding by polarity and trial arm**						**Total**
	**Negative**		**Neutral**		**Positive**		
	**Exp**.	**Cont**.	**Exp**.	**Cont**.	**Exp**.	**Cont**.	
**Context**	25	22	38	46	7	4	142
Factors relating to intervention	15	11	38	42	7	3	116
Factors relating to research method	10	11	0	4	0	1	26
**Implementation**	46	70	41	30	81	56	324
Delivery of tablet to PwD	1	2	0	2	13	11	29
Delivery of FMA app to PwD	6	n/a	5	n/a	4	n/a	15
Delivery of tablet/FMA app to CG	3	0	3	3	13	8	30
Investigator training with PwD	15	23	12	9	18	5	82
Investigator training with CG	7	25	9	11	15	16	83
Training and support PwD by CG	14	20	12	5	18	16	85
**Mechanisms of impact**	104	116	41	35	121	59	476
Usability—effectiveness	17	14	1	5	19	10	66
Usability—efficiency	12	18	3	2	15	4	54
Usability—user satisfaction	5	13	3	5	25	10	61
Usefulness	18	22	17	6	24	14	101
Learnability	25	30	14	13	23	11	116
Adoption	27	19	3	4	15	10	78

n/a, not applicable.

### 3.3. Context factors

#### 3.3.1. Quantitative data

A similar majority of people with dementia in both arms had some experience with a tablet before the study (experimental arm 58.3% of 12 responses; control arm 57.1% of 14 responses). A minority of caregivers in both arms reported that their partner could independently download apps before the project (experimental arm 21.4% of 14 responses; control arm 14.3% of 14 responses). The majority of caregivers in both arms, more in the experimental arm, reported having at least some experience with a tablet before the study (experimental arm 83.3% of 12 responses; control arm 63.6% of 11 responses).

#### 3.3.2. Qualitative data

Participants from both arms cited similar contextual factors. Their (partner's) prior experience with technology was often mentioned:

“*[My partner] also has a laptop but she doesn't use that much anymore. And we have a television… We don't really have any other devices.” [CG]*

“*He already used an iPad so he already has experience with a tablet.” [CG]*

Participants commented on contextual factors that had negatively impacted on their experience using the tablet, particularly citing limited time, other priorities, or decreasing motivation or capability of their partner:

“*Other things required more time and cost more energy. I'm not normally someone who keeps so many plates spinning… so I couldn't have the tablet on top of that.” [CG]*

“*She doesn't take the initiative to find things out or try things.” [CG]*

“*[She] is deteriorating rapidly. Both in terms of cognition and function, and motor skills. Now the carers are coming every day, and she's going three times a week to the day center.” [CG]*

Participants noted that changes to their routine due to illness or travel influenced how much they used the tablet, resulting either in more or less frequent use:

“*[My partner] has had corona and was very ill, so she has used the tablet less recently.” [CG]*

“*[We] have been on holiday. The tablet came with us but was used less.” [CG]*

“*[My partner] has corona and is in isolation. That means she's using the tablet more.” [CG]*

Participants were explicitly prompted to discuss financial or social factors which influenced tablet use but few commented on these topics. A few noted concerns about privacy when using apps, wanting to know what information might be collected by whom and why, but more frequently mentioned were the impact of a social event, such as a holiday, or how acceptable they found the idea of paying for apps:

“*[My partner] only used free apps but that wasn't to do with financial limitations, only that she was worried about being tied-in to something. If an app would be really useful for her, then we would be prepared to pay for it.” [CG]*

Investigators identified prior experience with a tablet as being an important factor in influencing participant experiences and outcomes:

“*The big difference is whether people already have experience with a tablet.” [INV]*

Hearing impairment was identified as a potential barrier to (online) contact during the study, but also a reason to prefer video call over telephone contact:

“*You have examples where it doesn't go so well because people can't hear you… on the other hand I noticed a few times that people with hearing impairment found video call easier than telephone because they could read lips.” [INV]*

Investigators also thought that caregivers' expectations to have played a role in their experience:

“*Sometimes caregivers expect a lot… or think that the person with dementia must be able to do a lot of things with the tablet for it to be a success… the caregiver can quickly get frustrated if that doesn't work out. On the other hand, setting expectations too low can also be demotivating.” [INV]*

### 3.4. Implementation factors

#### 3.4.1. Quantitative data

**Figure 2:**
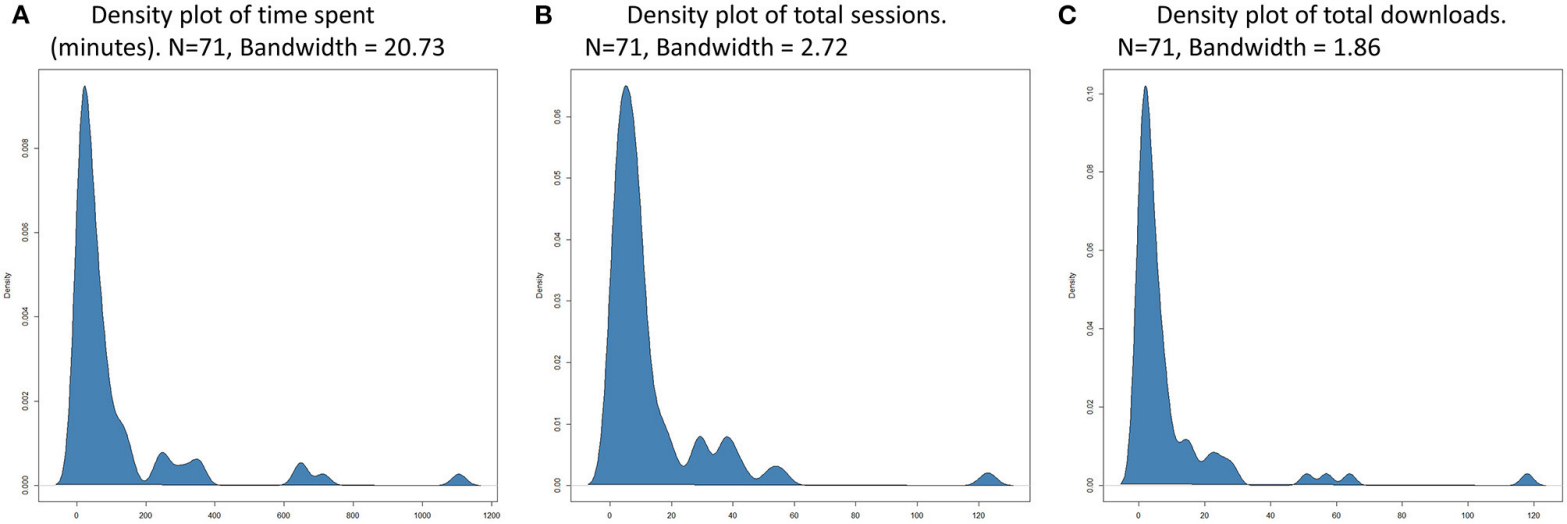
**(A)** Estimated kernel density of number of minutes spent using the FindMyApps app over 3 month intervention period. **(B)** Estimated kernel density of number of sessions using the FindMyApps app over 3 month intervention period. **(C)** Estimated kernel density of number of attempts to download apps via the FindMyApps app over 3 month intervention period.

Data from the caregiver proxy-report instrument regarding use of the tablet and automatically collected FindMyApps app usage data are described in Table 4, including test results of between-groups differences, which were not significant. Figure 2 presents estimated kernel density plots of the observed frequency and duration of FindMyApps app use, and number of attempted downloads, from automatically collected app-usage data, illustrating grossly non-normal distributions.

**Table 4:** Description of reported tablet and app use by participants, per trial arm and results of statistical tests for between group differences.

	**Experimental arm (*n* = 58)**	**Control arm (*n* = 61)**	**Test statistic**	**Significance**
	**Median (IQR) [range]**	**Median (IQR) [range]**		
Total score, reported frequency of tablet use	9 (5.0–10.8) [3–12]	9 (6–11) [3–12]	W = 1,875	*p* = 0.572
Total number of app-downloads reported	4 (1.3–7.0) [0–31]	2 (1–6) [0–25]	W = 1,534.5	*p* = 0.210
Total number of apps reportedly used	9 (4.3–14.0) [0–30]	9 (4–13) [0–41]	W = 1,673.5	*p* = 0.613
Observed total number of sessions with FindMyApps app	7 (3.5–13.0) [1–23]	n/a	n/a	n/a
Observed total minutes using FindMyApps app	43.6 (17.4–89.8) [1.5–1105.8]	n/a	n/a	n/a
Observed download attempts via FindMyApps	4 (1.5–8.0) [0–118]	n/a	n/a	n/a

n/a, not applicable.

Spearman's rho was calculated to investigate correlation between observed duration of FindMyApps app use and number of sessions (*n* = 57, rho = 0.83, *p* < 0.001) and between duration of FindMyApps app use and observed number of download attempts (*n* = 57, rho = 0.72, *p* < 0.001). Correlation was also calculated for complete cases between observed number of download attempts and proxy-reported number of downloads (*n* = 57, rho = 0.62, *p* < 0.001).

Negative binomial regression was used to further investigate differences between experimental and control arms (*n* = 118) regarding reported number of apps downloaded (rate ratio = 1.25, 95% CI 0.85–1.86) and reported number of apps used (rate ratio = 1.04, 95% CI 0.71–1.52). No relevant confounders were identified. Predictive multiple regression models for number of apps downloaded and used were constructed by backwards selection of variables with a cut-off of *p* = 0.10. The only significant predictor of the number of apps downloaded was whether the dyad cohabited (rate ratio for cohabiters = 1.69, 95% CI 1.05–2.70). For number of apps used, more apps were used by dyads who cohabited compared to those who did not (rate ratio = 1.78, 95% CI 1.12–2.82) and those with MCI rather than dementia (rate ratio = 1.51, 95% CI 1.02–2.26).

Of those completing the study, reported adherence to the advised minimum of at least two practice sessions a week for the first 4 weeks was 62.5% in both arms. In the experimental arm, 63.1% reported downloading at least one app, compared to 67.6% in the control arm.

Similar proportions of respondents with dementia from both arms reported adequate support from their partner in using the tablet (experimental arm 91.7% of 12 responses; control arm 84.6% of 13 responses). All investigators agreed that it was easy to communicate with participants during the online training, that video call is an appropriate method for the training and that the FindMyApps app mostly functioned properly. Two of three investigators had succeeded in providing training by video call in all cases.

#### 3.4.2. Qualitative data

The content of comments between trial arms did not substantially differ. Regarding the technological components of the intervention, most participants were positive:

“*No, no technical problems. None at all. I've had this iPad two or three months now and it works perfectly… the things I wanted to do [with the FindMyApps app] went well.” [PWD]*

Of the few negative comments, several related to the speed of the FindMyApps app, or their own internet connection for example:

“*The app functioned quite slowly.” [PWD]*

“*[We] tried to video call family and ex-colleagues. It didn't work, the quality of the picture was poor.” [CG]*

Regarding the online training, participants in the experimental arm particularly noted a personalized, step-wise approach, commenting more positively about the experience than control arm participants, for example:

“*What I really thought was good about the training – and it really worked for my mother – was that [the trainer] explained everything first, and then immediately gave the instruction for her to do it herself. And she was able to do it immediately.” [CG]*

Primarily in the control arm, participants stated that they would have preferred face-to-face training, and more frequently repeated:

“*Perhaps the training should be in real life, then you can also ask questions more easily.” [PWD]*

“*It would have been better if [the trainer] had gone round three times or so in the first week.” [CG]*

“*Yes, maybe it's useful to check after a week or two if it's working. You say we can always call if there are problems, but we're not so quick to do that.” [CG]*

One reason given for needing face-to-face and more frequent training was limited concentration of the person with dementia:

“*It's already very difficult for my husband to concentrate on [the training].” [CG]*

In both groups, many participants were positive about training by video call, especially compared to communication by telephone:

“*I found it an easy way to call. Nice to see each other, I think you have better contact that way.” [PWD]*

“*I found the video call much better than this [phone call]!” [PWD]*

Investigators commented that the technology could be unpredictable. The quality of the internet connection was an important potential barrier, beyond the investigator or participant's control:

“*The unpredictability of tablets and technology can make it difficult… you don't always know what's causing something.” [INV]*

“*You always have problems which can crop up… like a slow internet connection.” [INV]*

Where participants used their own tablets, certain settings could also cause unexpected challenges:

“*I had someone with really large text on a small tablet. Everything was out of balance and difficult to read. That was difficult.” [INV]*

With respect to the training, investigators noted limits to what could be achieved by video call and a single session:

“*I think [video call] is a very good alternative [to face-to-face] if it's about learning to use an app… for teaching someone to use the device itself I think it's less suitable.” [INV]*

“*Another tip would be splitting the training into two sessions… many participants found it a lot of information all in one go.” [INV]*

The support of caregivers helping the person with dementia to learn to use the tablet was felt to be of highly varying quality:

“*I got the idea that [extra training was needed] if the caregiver wasn't competent or was impatient… that didn't help the learning process.” [INV]*

“*You notice that if the person with dementia and caregiver both find [using the tablet] difficult then tension can build between them.” [INV]*

### 3.5. Mechanisms of impact factors

#### 3.5.1. Quantitative data

**Figure 3:**
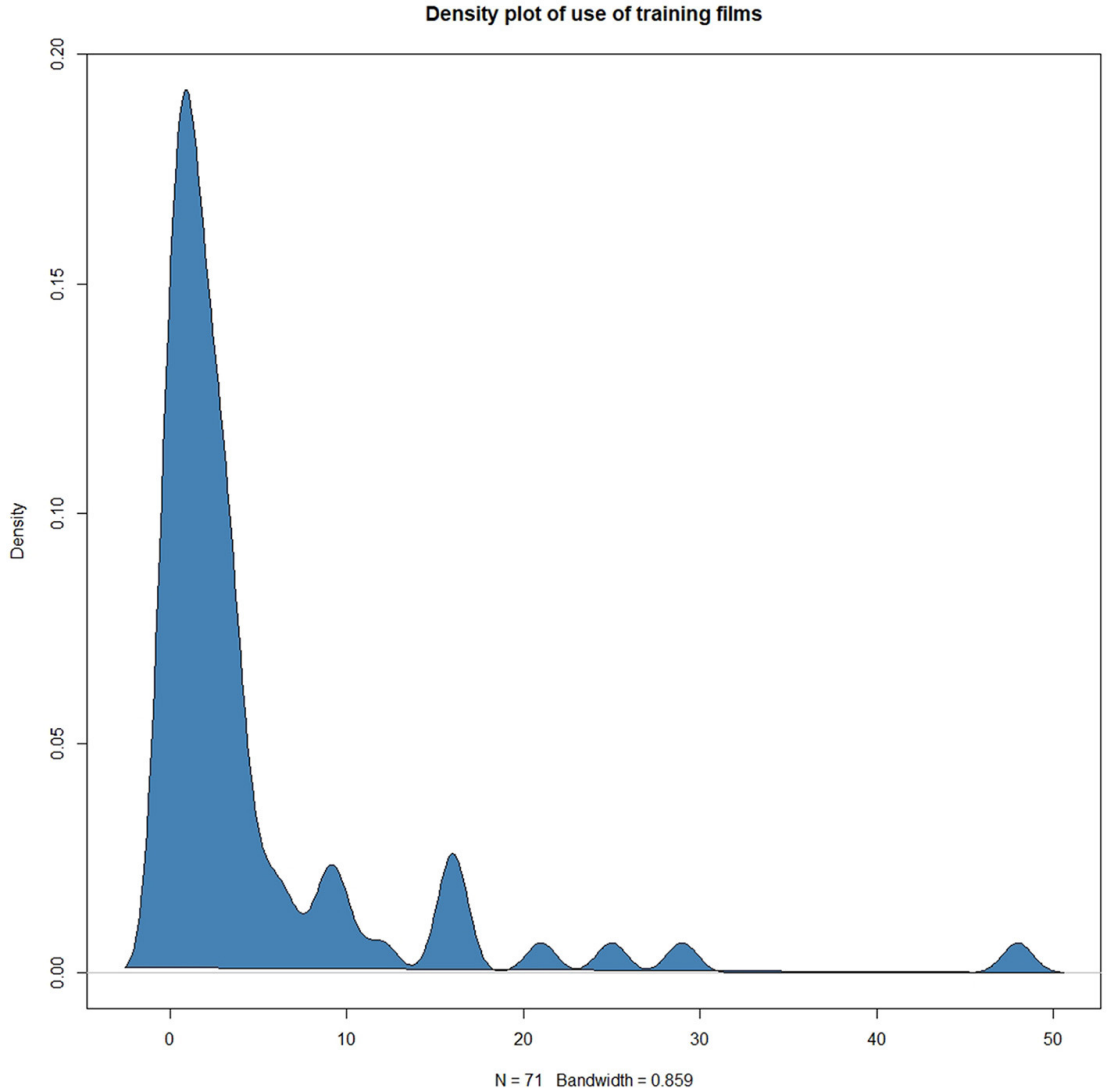
Estimated kernel density plotted against number of clicks to access in-app training films by FindMyApps users during a 3 month intervention period.

**Figure 4:**
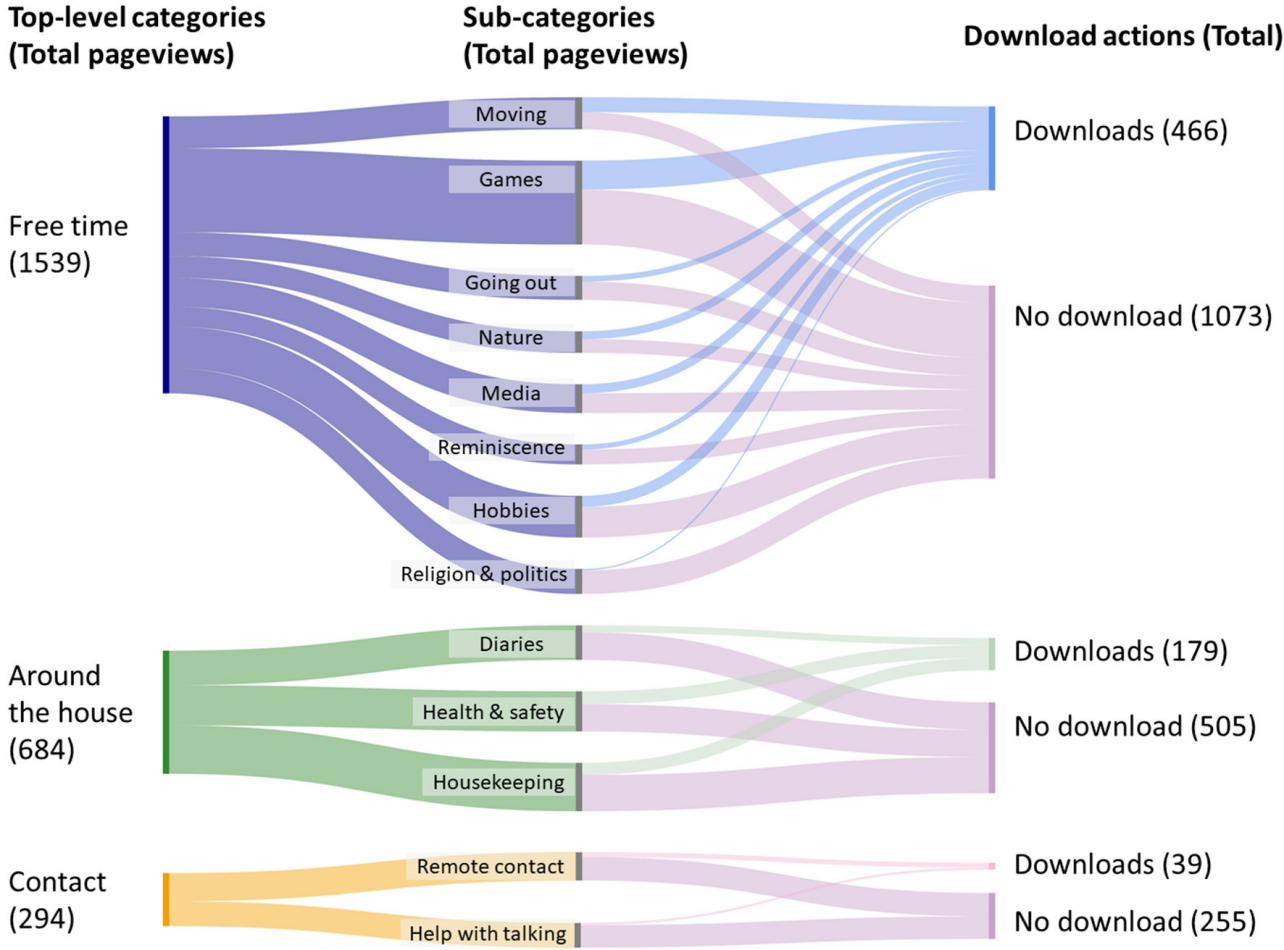
Sankey diagram visualizing aggregated user flows, from viewing one of the top-level app categories **(on the left)**, proceeding to view sub-categories **(in the middle)**, to terminating the search with or without downloading an app.

Figure 3 shows estimated kernel density of the number of times users of the FindMyApps app clicked to access the in-app training films during the 3-month intervention period. Figure 4 shows the frequencies with which each category and sub-category of the FindMyApps database were viewed by users, and with which users attempted to download apps from these categories.

Responses to closed questions relating to mechanism of impact factors from the SSIs with RCT participants are presented in Table 5. The following trends were noted in the experimental arm compared to control arm: higher mean scores (and lower SDs) given by people with dementia regarding how useful, easy to use, and fun the tablet was; higher proportion of caregivers reporting that their partner learned to download apps use the tablet independently; and higher proportion of people with dementia reporting that the tablet was easy or very easy to learn to use, and that the instruction films helped them. All investigators interviewed disagreed that a one-off training session was always sufficient for participants to make further progress in learning to use the tablet by themselves.

**Table 5:** Summary of answers to closed questions posed during SSIs—mechanism of impact factors.

	**Experimental arm**			**Control arm**		
	**Response** ***n***	**Mean (SD)**	**Percent agreeing**	**Response** ***n***	**Mean (SD)**	**Percent agreeing**
**Person with dementia**						
How useful was the tablet? (0–10)	10	7.5 (1.3)	n/a	10	6.5 (1.9)	n/a
How easy to use was the tablet? (0–10)	11	7.6 (0.9)	n/a	12	5.5 (2.1)	n/a
How fun to use was the tablet? (0–10)?	11	7.0 (2.6)	n/a	11	5.8 (2.3)	n/a
How easy to use was the FindMyApps app (0–10)? (E)	9	7.3 (0.5)	n/a	n/a	n/a	n/a
How fun to use was the FindMyApps app? (0–10) (E)	9	6.6 (2.7)	n/a	n/a	n/a	n/a
I found it easy or very easy to learn to use the tablet	10	n/a	90.0	11	n/a	27.3
I found it easy or very easy to find an app I wanted with FindMyApps (E)	8	n/a	100.0	n/a	n/a	n/a
I found it easy or very easy to learn to use the FindMyApps app (E)	8	n/a	100.0	n/a	n/a	n/a
I can download apps by myself, using FindMyApps (E)	11	n/a	63.6	n/a	n/a	n/a
I can download apps by myself (C)	n/a	n/a	n/a	13	n/a	30.8
The instruction films helped me learn to use the tablet and FindMyApps app (E)	11	n/a	45.5	n/a	n/a	n/a
The instruction films helped me learn to use the tablet (C)	n/a	n/a	n/a	11	n/a	0.0
**Caregiver**						
How useful was the tablet? (0–10)	14	6.9 (2.2)	n/a	12	6.4 (2.3)	n/a
How easy to use was the FindMyApps app (0–10)? (E)	13	7.4 (1.3)	n/a	n/a	n/a	n/a
How easy to use was the tablet (0–10)? (C)	n/a	n/a	n/a	11	7.3 (2.0)	n/a
How fun to use was the FindMyApps app? (0–10) (E)	12	5.7 (3.0)	n/a	n/a	n/a	n/a
How fun to use was the tablet? (0–10) (C)	n/a	n/a	n/a	12	7.2 (1.6)	n/a
My partner can sometimes or always download apps by themselves now	14	n/a	42.8	14	n/a	14.3
My partner can sometimes or always use the tablet by themselves	14	n/a	64.2	14	n/a	42.9
My partner can sometimes or always use the FindMyApps app by themselves (E)	13	n/a	46.2	n/a	n/a	n/a

Questions marked (E) were only asked of experimental arm participants, questions marked with (C) were only asked of control arm participants. n/a, not applicable.

#### 3.5.2. Qualitative data

##### 3.5.2.1. Effectiveness, efficiency and user satisfaction

Participants in the experimental arm particularly reported being pleased that they could easily find apps that they enjoyed using:

“*We found apps via FindMyApps. For example, the app with old photos of Amsterdam. Without FindMyApps we'd never have happened across that. So that was really great.” [CG]*

“*It's easier to search because of the categories and pictograms, which means she can search more purposefully... There's also a limited selection, which makes it less cluttered than the App Store. It's easier if you have fewer choices.” [CG]*

“*I think it's really fun, everyone should discover these apps.” [PWD]*.

Similar themes were raised by participants in the control arm, however, with respect to difficulties and inefficiencies, which reduced satisfaction:

“*Searching for simple things went ok. But sometimes so many apps appear that you don't know how to deal with it… Sometimes I can't see the wood for the trees anymore.” [PWD]*

“*You have to click a lot of times before you can see the apps, there are lots of apps on the list which my wife won't use.” [CG]*

“*At some point it all gets too complicated and that's frustrating.” [CG]*

Participants from both groups found that the presence of in-app advertising reduced satisfaction and efficiency, for example:

“*[My partner] struggles with adverts, she gets confused by them. I tried to get rid of [apps with] adverts but it's difficult.” [CG]*

##### 3.5.2.2. Usefulness

In the experimental arm, many of the positive comments about usefulness focused on particular characteristics of the tablet and apps, such as the portability and size, relative to a laptop and mobile phone, respectively:

“*You wouldn't take the laptop on holiday, but you would take the tablet. And you can take photos with it. Very handy.” [PWD]*

“*Very useful. I take the tablet everywhere I go. The size is better than a mobile phone.” [PWD]*

Negative comments reflected that whilst the tablet was suited to portability, it was inferior to a computer for use at home, especially during the COVID-19 pandemic:

“*We rarely leave the house. Previously we took the iPad with us if we went somewhere. Now everything happens at home so the computer is better. The tablet actually hasn't been used.” [CG]*.

“*The tablet wasn't useful, as such… I haven't seen any sign of that yet.” [PWD]*

There were also comments that certain features of the FindMyApps app, such as the ability to update user preferences, had not been needed or used:

“*The preferences weren't changed. That was all good.” [CG]*

Comments in the control arm followed similar themes. An example of a positive comment was:

“*I can certainly see the advantages of using a tablet.” [PWD]*

An example of a negative comment was:

“*I can play chess and listen to music with YouTube on my laptop. So, I didn't really see the need for something else on top of that.” [PWD]*

##### 3.5.2.3. Learnability

In the experimental arm, positive comments regarding learnability frequently concerned practice effects, for example:

“*It's definitely easier now, because of practicing.” [PWD]*

An example of a negative comment was:

“*My father can't learn new things anymore like we can.” [CG]*

In the control arm, there were more negative comments, following similar themes to the experimental arm such as:

“*It should all be made as simple as possible. The handbook was still too complicated for my wife.” [CG]*

“*I found it difficult to learn to use the tablet and I don't think that's going to change.” [PWD]*

Very few participants from either arm made use of the helpdesk facility but the comments from those who had were positive, for example:

“*I got in touch once because the handbook was missing and [another copy] was sent very quickly.” [CG]*

##### 3.5.2.4. Adoption

With respect to adoption of the intervention there were more negative comments in the experimental arm, compared to the control arm. Participants commented that they had used the FindMyApps app but not on a regular basis, or less than other tools:

“*I used [the FindMyApps app] mostly at the beginning.” [PWD]*

“*I do most things with my phone, and occasionally I use the laptop. I actually don't use the tablet.” [PWD]*

Whilst there were fewer positive responses, some participants were very enthusiastic adopters:

“*In the beginning I didn't use any apps at all, and now every day.” [PWD]*

In the control arm there were similar negative and positive comments regarding adoption:

“*…it's not part of my daily routine… I don't understand yet what I can do with it. I do more with a laptop.” [PWD]*

“*I use the tablet a lot in my day-to-day life. For almost everything.” [PWD]*

Investigators particularly commented that the helpdesk was rarely used, and related this to broader aspects of learnability:

“*[The helpdesk was used] very little, I think... two or three participants with technical questions… I had one participant who called more often… they got in touch around once per week.” [INV]*

“*It's important that the person with dementia is prepared to ask for help from those around them.” [INV]*

### 3.6. Triangulation of key insights

Results of triangulation of key insights from the proxy-reports, analytics data and SSIs are presented in Table 6.

**Table 6:** Triangulation of key insights on facilitators and barriers from all data sources, concerning context, implementation and mechanism of impact factors which may have impacted on expected behavioral outputs of the FindMyApps intervention and therefore outcomes of the ongoing RCT.

	**Finding**	**CGPRI**	**AAUD**	**SSI-P**	**SSI-I**
Context	Previous experience of the caregiver and person with dementia with digital technology perceived to have facilitated learning, whereas lack of any experience made learning more difficult	+	0	+	+
	Caregivers in both arms reported insufficient time to implement the intervention, due to other priorities	+	0	+	0
	Caregivers experience apathy and lack of motivation from person with dementia experienced as barriers	+	0	+	0
	Caregivers' expectations regarding rate of learning and capacity of person with dementia to use the tablet independently may have been a barrier if expectations not met	0	0	0	+
Implementation	Generally few technical problems encountered, though slow Wi-Fi connections were a barrier	+	0	+	+
	**Conducting training by video call was possible in virtually all cases**, though those without any previous tablet experience may have found it less valuable than a face-to-face session	+	0	+	+
	Experimental arm participants reported more positive experience of the training, citing personalized and stepwise approach, whereas control arm participants reported more negative experience of training	+	0	+	0
	Most people with dementia reported receiving adequate support from their caregiver with the tablet	+	0	+	-
	Investigators felt that quality of support provided by caregivers to people with dementia was an important source of variability	0	0	–	+
	**Majority of participants, around two in three study completers, adhered to advised frequency of tablet practice**	+	+	0	0
	**Most participants, around two out of three in each arm, downloaded at least one additional app following supervised practice session, and in experimental arm this was associated with use of FMA**	+	+	0	0
	**Trend, not statistically significant, of experimental arm participants downloading more apps (median 4 vs. 2)**	+	0	0	0
	Three clusters of intensity of FMA use: most made little use, few made moderately intense use, even fewer made more intense use	0	+	0	0
Mechanisms of impact	**Most participants made little use of training or support opportunities, a few made frequent use**	+	+	+	+
	Frequency of views of different app categories and sub-categories in FMA and respective ratios of views to download attempts varied greatly	0	+	0	0
	**Experimental arm participants made more positive comments about how useful, easy to use, fun and easy to learn the intervention was, whereas control arm participants made more negative comments**	+	0	+	0
	In both arms, barriers to using downloaded apps were reported, most frequently citing pop-up adverts	+	0	+	0
	In both arms, the added value of the tablet was experienced to be as a portable tool for use away from home	+	0	+	0
	**Generally little to moderate adoption of tablet use, with a few very enthusiastic participants**	+	0	+	0
	In experimental arm, FMA generally reported to be used initially to find apps, thereafter much less	+	0	+	0

CGPRI, caregiver proxy-report instrument; SSI-P, semi-structured interview with trial participants; SSI-I, semi-structured interview with investigators; AAUD, automatically collected app-usage data; FMA, FindMyApps app; “+”, supports finding; “0”, silent on finding; “–”, contradicts finding. Findings in bold relate directly to observation of the expected behavioral outputs of the FindMyApps intervention.

## 4. Discussion

### 4.1. Principal results

This process evaluation identified context, implementation and mechanism of impact factors which may have influenced the anticipated behavioral outputs of the FindMyApps intervention and therefore the outcomes of the ongoing RCT. Participants did engage with training sessions by video call, though subsequently made less use than expected of available learning and support resources (instruction films and telephone helpdesk). The majority of participants adhered to the advised training schema, searched for and downloaded apps, which in many cases reportedly met their needs and interests, and there were reports that participants did improve their tablet skills through practice. There was mixed evidence for adoption of the use of tablet-based apps, with a small number of users being very positive, and a larger group being less enthusiastic. Reported and observed behavioral outputs of the experimental and control arm participants were largely similar. There was a trend that experimental arm participants reported more app downloads, but not quantitatively more app use.

A number of facilitators and barriers were further identified, which may have influenced the above behavioral outputs, and therefore may also influence the outcomes of the ongoing RCT.

#### 4.1.1. Context factors

Evidence from multiple data sources suggested that those with no previous tablet experience faced greater difficulties in learning to use the tablet. This is in line with results from previous studies of FindMyApps and other interventions for this target group (16, 18, 31, 36–38). Those who cohabited with their caregiver and those with a diagnosis of MCI also tended to download and/or use more apps. These findings validate the decision to stratify randomization on exactly these three variables. Lack of time, other priorities and apathy or lack of motivation of the person with the dementia were consistently reported by caregivers as barriers. Indeed, both high caregiver burden and apathy are well-documented in dementia (2, 39). These may be the most important reasons why around 1 in 3 participants did not adhere to the advised training schema.

#### 4.1.2. Implementation factors

Aside from slow Wi-Fi connections, few technical problems were experienced. This may be one reason why participants felt little need to use the telephone helpdesk. Training provided by video call was more valuable for those with tablet experience and training received by experimental arm participants was more positively experienced, suggesting that the FindMyApps training met participants' needs. There were conflicting views on the support that people received from caregivers during the study: people with dementia themselves consistently rated the support positively, whereas investigators felt that the quantity and quality of support was an important source of variation in outcomes. This could reflect some social desirability bias on the part of the people with dementia, since the relationship with their caregiver (in most cases a spouse) is loaded with social norms (40).

#### 4.1.3. Mechanism of impact factors

That users in the experimental arm were generally positive about how easy to use, easy to learn, useful and fun the intervention was might offer another explanation for why they made less use than expected of training opportunities, and for the trend for experimental arm participants to download more apps. FindMyApps users more frequently searched for and downloaded apps relating to “free time”, fun activities compared to apps that might support instrumental activities of daily living, or apps for social contact. This is in line with previous findings about how older users, particularly those with dementia, perceive and use technology, namely that, “persons with dementia value the potential of technology to have fun and pleasure with it” (41). In the case of social contact, this may also be because the tablets had apps for video-calling and instant messaging pre-installed. In relation to the primary outcomes of the RCT, these results might imply a higher likelihood of demonstrating an effect of FindMyApps on self-management than on social participation. However, there is insufficient data from this process evaluation on how apps were used, and how effective the apps were, to draw strong conclusions. Indeed, participants in both arms reported limitations of apps used, particularly related to pop-up advertising, and limitations of the utility of the tablet itself, being primarily valuable for its portability. The fact that lockdowns to prevent spread of COVID-19 were in place for much of the data collection period might explain hesitancy reported regarding adoption.

### 4.2. Strengths and limitations

The use of mixed methods, to collect and analyze both quantitative and qualitative data allowed for a comprehensive exploration of behavioral outputs and factors which may have impact on the outcomes of the ongoing RCT. There were still limitations to the data collected. Self and proxy-report data were collected after 1, 2, and 3 months, whereas the future use of methods such as ecological momentary assessment or experience sampling might allow for data collection with higher temporal resolution and less recall bias (42, 43). Due to privacy measures taken by Google and Apple, it was not possible to directly observe the use of apps other than the FindMyApps app. Collection of pseudonym zed data would have strengthened this study by allowing between-group analyses of observed tablet use, in addition to reported use. With respect to generalizability of our findings, participation in the study was voluntary and the study sample is expected to be biased toward people with a particular interest in research or technology, or who were otherwise willing and able to participate. For example, over 50% of people with dementia in this study had completed higher education, compared with around 32% of the Dutch population aged over 55 (44). Future research focusing on large-scale implementation should seek to evaluate the intervention with a more representative sample.

### 4.3. Comparison with prior work

Almost all insights from the SSIs confirm results of a previous, smaller-scale process evaluation with FindMyApps, which in turn was largely in line with findings from feasibility studies during development of the intervention (16, 31). The main differences were fewer technical problems with the FindMyApps app, and fewer negative experiences of the training, particularly in the experimental arm. Since the previous study, the app has been upgraded from a web-app to a native app, which likely explains the reduction in technical problems. The improved experience of training could be due to improvements made between the pilot and definitive trials, in the training standard operating procedure. However, a more substantial difference from the pilot trial was moving from face-to-face training to online training by video call (due to COVID-19 related restrictions). Our findings therefore contrast with results from an earlier Dutch study evaluating an online intervention for caregivers of people with dementia, which found a preference for hybrid over online-only contact (45). It may be that attitudes and skills of the target group with respect to technology have changed over time, with some evidence suggesting adaptation to COVID-19 lockdowns has spurred this development (46). Findings with respect to sampling bias, adherence and factors which may impact outcomes are in line with earlier studies of other eHealth interventions for people with dementia and their caregivers (36–38). Lower adoption of tablet-based apps than might be expected based on practical skills and indicators such as access to the internet has also been anticipated in the literature (47).

### 4.4. Scientific and practical relevance and recommendations for research and practice

The results of this study demonstrate the value of mixed methods process evaluations, to accompany RCTs evaluating the effectiveness of eHealth interventions. There are several implications of these results for the ongoing FindMyApps RCT. The quality of trial participants' interactions with the tablet and downloaded apps is more likely to be the source of any effect on outcomes, than the quantity. The effect of the intervention may have been large on a small number of participants, and small on a large number of participants, and since the trial is powered to detect on average a moderate effect size of the intervention, this may not be sufficient to detect an overall effect. Where possible the effect of the identified factors on the outcomes of the ongoing RCT should be investigated by *post-hoc* analyses, for example, whether the person with dementia was reported to be experiencing apathy at baseline. With respect to future implementation of FindMyApps, several recommendations can be made, based on the facilitators and barriers mentioned by people with dementia and caregivers. Collectively, the following improvements might lead to more sustainable adoption beyond 3 months: additional support and the option for face-to-face training should be provided, particularly for those with no previous tablet experience; additional training sessions provided by professionals within the first 4 weeks may help to reduce caregiver burden and improve adherence; the selection of apps in the most frequently viewed categories, and in categories with large numbers of views but few downloads should be expanded, if possible; and the selection of apps without pop-up advertising should be expanded. Future research accompanying larger-scale implementation of FindMyApps should be undertaken, to test whether these improvements to the intervention indeed lead to higher rates of adoption, and to understand and be able to predict which categories of apps are most interesting to users based on their background characteristics.

## 5. Conclusion

FindMyApps seems to have had more impact on the quality of interactions with tablet apps, than on the quantity of interactions. Factors related to context, implementation and mechanisms of impact which may have influenced the behavior of participants and therefore may affect self-management and social participation outcomes should be considered when interpreting the RCT results. Whilst this study improved on previous evaluations of digital interventions for people with dementia, future studies of eHealth interventions should aim to achieve more representative samples. Future development and implementation of digital interventions should take account of supporting users unfamiliar with digital technology in order to improve adoption rates.

## Data availability statement

The raw data supporting the conclusions of this article will be made available by the authors, without undue reservation. Those seeking to access the field note transcripts of the interviews, or any of the analytics data can contact DN, d.n.neal@amsterdamumc.nl, to request redacted transcripts or other data.

## Ethics statement

The studies involving human participants were reviewed and approved by the Medical Ethics Committee of the VU Medical Centre. The patients/participants provided their written informed consent to participate in this study.

## Author contributions

DN, TE, KD, and R-MD: conceptualization. DN and R-MD: methodology. DN, LK, DP, CO, SN, TE, and R-MD: formal analysis. DN, LK, DP, CO, and SN: investigation. R-MD: resources and funding acquisition. DN, LK, and DP: data curation. DN: writing—original draft preparation, visualization, and project administration. DN, LK, DP, CO, SN, TE, KD, MM, and R-MD: writing—review and editing. TE, KD, MM, and R-MD: supervision. All authors have read and agreed to the published version of the manuscript. All authors contributed to the article and approved the submitted version.
