# Supplementary material for: FindMyApps eHealth intervention improves quality, not quantity, of home tablet use by people with dementia
Source: Front Med (Lausanne). 2023 May 30;10:1152077. doi: 10.3389/fmed.2023.1152077 (PMC10262310; doi:10.3389/fmed.2023.1152077)
Supplement: Supplementary file 1 [file Data_Sheet_1.docx]

Supplementary Material

FindMyApps eHealth intervention improves quality, not quantity, of home tablet use by people with dementia

**David P. Neal*, Leanne Kuijper, Daniela Pistone, Channah Osinga, Sanne Nijland, Teake Ettema, Karin Dijkstra, Majon Muller, Rose-Marie Dröes**

*** Correspondence:** David P. Neal: d.n.neal@amsterdamumc.nl

# Supplementary Material: Interview guides

#

The following versions of the interview guides were translated from the original Dutch by David Neal.

# SSI Person with dementia –Control Group – V7.0

Name of interviewer:

Code PwD and CG:

Date and time:

| **A. Implementation of the intervention for PWD** | | |
| --- | --- | --- |
|  | **Did the tablet work correctly during the research, as far as you’re concerned?**  Possible follow-up questions:   - Did you have problems turning the tablet on? - Did you have problems with the internet connection?   Answer likely to fall under code A1(i) . | ☐ Yes ☐No |
|  | **To what extent do you agree with the following statement:**  **My caregiver provided sufficient support to be able to use the tablet**  Can you tell me something about how you experienced the support from your caregiver?  Answer likely to fall under code A3 . | ☐Completely disagree ☐ Somewhat disagree ☐ Neither agree nor disagree  ☐ Somewhat disagree ☐Completely agree  ☐ I didn’t need support |
|  | **How did you experience the online training at the start of the research project?**  Possible follow-up questions:   - Could you follow the online training? - Did you find the training useful? - Did the level and topics meet your needs? - Did you succeed in applying what you learned? |  |
|  | **Do you have suggestions for improving the training, for example, additional topics that you missed?** |  |
| **B. Mechanisms of impact for PWD**  **B1. Usability** | | |
|  | **Can you tell me something about your experience with the tablet?**  Possible follow-up questions:   - Did you find the tablet easy to use? - Was the tablet easier or more difficult to use than your laptop/phone? Why? - Did you enjoy using the tablet?   Answer likely to fall under code B1. |  |
|  | **What grade would you give the tablet in terms of general usability?**  Answer likely to fall under code B1 and/or B3. | 1. ..1 ..2 ..3 .. 4 .. 5 .. 6 .. 7.. 8 .. 9 .. 10 |
|  | **What grade would you give the tablet in terms of how much fun you had with it?**  Answer likely to fall under code B1(iii). | 1. ..1 ..2 ..3 .. 4 .. 5 .. 6 .. 7.. 8 .. 9 .. 10 |
| **B2. Usefulness** | | |
|  | **In which ways did the tablet support you?**  Wait for answer. Having noted the answer, prompt with the following options:   - Self-management? - Meaningful activities? - Social contact?   Answer likely to fall under code B2 . | ☐Self-management ☐Meaningful activities ☐ Social contact ☐Other |
|  | **What grade would you give the tablet in terms of how useful it was?**  Answer likely to fall under code B2. | 1. ..1 ..2 ..3 .. 4 .. 5 .. 6 .. 7.. 8 .. 9 .. 10 |
| **B3. Learnability of tablet** | | |
|  | **How did you experience learning to use the tablet? Can you tell me something about that?**  Answer likely to fall under code B3 . | ☐Difficult ☐Somewhat difficult ☐Easy ☐ Very easy |
|  | **Were you able to download apps yourself? (with or without help from your partner)**  Answer likely to fall under code B3 . | ☐Yes ☐No |
|  | **Did the instruction films help you to learn to use the tablet?**  Possible follow-up questions: If not, why not?  Answer likely to fall under code B3 . | ☐Yes ☐No |
|  | **Do you have suggestions to make learning to use the tablet easier?**  Answer likely to fall under code B3 . |  |
| **B4. ADOPTION of tablet by PWD** | | |
|  | **To what extent is using the tablet part of your daily routine now?**  Answer likely to fall under code B4 . |  |
| **C. Contextual factors** | | |
|  | **Before you started this research, what other technology or devices did you use regularly?**  Wait for answer before prompting with options  Answer likely to fall under code C | ☐ None/nothing ☐ Computer or laptop ☐ Smartphone (e.g. iPhone)  ☐ Other, namely: |
|  | **Before you started this research, did you already have experience using a tablet?**  Answer likely to fall under code C | ☐ Yes ☐ A little bit ☐ No |
| **GENERAL OBSERVATIONS** | | |
| / | How long did the interview last? |  |
| / | Other important contextual information? |  |

# SSI for the caregiver –Control Group – V1.0

Name interviewer:

Code PwD and CG:

Date and time:

| 1. **Implementation** | | |
| --- | --- | --- |
|  | **Did the tablet work correctly during the research, as far as you’re concerned?**  Possible follow-up questions:   - Did you have problems turning the tablet on? - Did you have problems with the internet connection?   Answer likely to fall under code A1 |  |
|  | **How did you experience the online training at the start of the research project?**  Possible follow-up questions:   - Could you follow the online training? - Did you find the training useful? - Did the level and topics meet your needs? - Did you succeed in applying what you learned?   Answer likely to fall under code A2 |  |
|  | **Do you have suggestions for improving the training, for example, additional topics that you missed?**  Answer likely to fall under code A2 |  |
|  | **What was your experience of calling the ‘helpdesk’ for support?**  Answer likely to fall under code A1 | ☐ Not used |
|  | **How did you find it to explaining the functions of the tablet to your partner?**  Answer likely to fall under code B2 |  |
|  | **Did the training help you to support your partner to learn to use the tablet?**  Answer likely to fall under code B2 |  |
| **B. Mechanisms of Impact**  **B1. Usability** | | |
|  | **Which barriers did you encounter in using the tablet, or what made using the tablet difficult?**  Answer likely to fall under code B1 |  |
|  | **What grade would you give the tablet in terms of general usability?**  Answer likely to fall under code B1(i and ii) and/or B3 . | 1. ..1 ..2 ..3 .. 4 .. 5 .. 6 .. 7.. 8 .. 9 .. 10 |
|  | **What grade would you give the tablet in terms of how much fun you had with it?**  Answer likely to fall under code B1(iii). | 1. ..1 ..2 ..3 .. 4 .. 5 .. 6 .. 7.. 8 .. 9 .. 10 |
| **B2. Usefulness** | | |
|  | **What grade would you give the tablet in terms of how useful it was?**  Answer likely to fall under code B2. | 1. ..1 ..2 ..3 .. 4 .. 5 .. 6 .. 7.. 8 .. 9 .. 10. |
| **B3. Learnability** | | |
|  | **Was your partner already able to download apps by themselves before this research?**  Answer likely to fall under code B3 | ☐Yes ☐ Sometimes ☐No ☐Not attempted |
|  | **Can your partner download apps by themselves now?**  Answer likely to fall under code B3 | ☐Yes ☐ Sometimes ☐No ☐Not attempted |
|  | **Can your partner use the tablet by themselves?**  Follow-up:  **Is your partner more or less proficient in using the tablet than before?**  Answer likely to fall under code B3 | ☐Yes ☐ Sometimes ☐No ☐Not attempted |
|  | **Do you have suggestions to make learning to use the tablet easier?**  Answer likely to fall under code A, B1 and B3 |  |
| **B4. Adoption** | | |
|  | **To what extent is using the tablet part of your daily routine now?**  Answer likely to fall under code B4 . |  |
| **C. Contextual Factors** | | |
|  | **Before you started this research, what other technology or devices did you use regularly?**  Wait for answer before prompting with options (e.g. laptop, smartphone, etc.)  Answer likely to fall under code C |  |
|  | **Before you started this research, did you already have experience using a tablet?**  Answer likely to fall under code C | ☐ Yes ☐ A little bit ☐ No |
|  | **Did material, social or financial factors influence your use of the tablet or choice of apps?**   - Material factors include for example features of the home - Socal environment includes cohabiting with partner, for example   Answer likely to fall under code C |  |
| **GENERAL OBSERVATIONS** | | |
| / | How long did the interview last? |  |
| / | Other important contextual information? |  |

# SSI Person with dementia – Experimental Group – V7.0

Name of interviewer:

Code PwD and CG:

Date and time:

| **A. Implementation of the intervention for PWD** | | |
| --- | --- | --- |
|  | **Did the tablet work correctly during the research, as far as you’re concerned?**  Possible follow-up questions:   - Did you have problems turning the tablet on? - Did you have problems with the internet connection?   Answer likely to fall under code A1(i) . | ☐ Yes ☐No |
|  | **Did the FindMyApps app work correctly during the research, as far as you’re concerned?**  Possible follow-up questions:   - Did you have problems launching the FindMyApps app? - Did you have problems logging into the FindMyApps app?   Answer likely to fall under code A1(ii) . | ☐ Yes ☐No |
|  | **To what extent do you agree with the following statement:**  **My caregiver provided sufficient support to be able to use the tablet and the FindMyApps app.**  Can you tell me something about how you experienced the support from your caregiver?  Answer likely to fall under code A3 . | ☐Completely disagree ☐ Somewhat disagree ☐ Neither agree nor disagree  ☐ Somewhat agree ☐Completely agree  ☐ I didn’t need support |
|  | **How did you experience the online training at the start of the research project?**  Possible follow-up questions:   - Could you follow the online training? - Did you find the training useful? - Did the level and topics meet your needs? - Did you succeed in applying what you learned? |  |
|  | **Do you have suggestions for improving the training, for example, additional topics that you missed?** |  |
| **B. Mechanisms of impact for PWD**  **B1. Usability** | | |
|  | **Can you tell me something about your experience with the tablet?**  Possible follow-up questions:   - Did you find the tablet easy to use? - Was the tablet easier or more difficult to use than your laptop/phone? Why? - Did you enjoy using the tablet?   Answer likely to fall under code B1 . |  |
|  | **What grade would you give the tablet in terms of general usability?**  Answer likely to fall under code B1 and/or B3. | 0 ..1 ..2 ..3 .. 4 .. 5 .. 6 .. 7.. 8 .. 9 .. 10 |
|  | **What grade would you give the tablet in terms of how much fun you had with it?**  Answer likely to fall under code B1(iii). | 0 ..1 ..2 ..3 .. 4 .. 5 .. 6 .. 7.. 8 .. 9 .. 10 |
| **The following questions are specifically about the FindMyApps app** | | |
|  | **Did you use the FindMyApps app (to search for new apps)?**  **Can you tell me something about your experience with the FindMyApps App?**  Possible follow-up questions:   - Could you find apps which met your interests and needs? [B1(i)] - How easy or difficult was it to use the FindMyApps app to search for apps? [B1(ii)] - What kind of apps or topics were missing, do you think? [B1(i)/B1(iii)] - Did you find it fun to search for new apps with the FindMyApps app? [B1(iii)]   Answer likely to fall under code B1(i-iii) . |  |
|  | **Did you use the following features of the FindMyApps app:**  Answer likely to fall under code B1(i-iii) and B3 . |  |
|  | **The personal preferences which you (with help from your caregiver) could change?**  If yes, could you tell me something about your experience with this feature?  If not, why not? | ☐ Yes ☐No |
|  | **The page ‘My Apps’**  If yes, could you tell me something about your experience with this feature?  If not, why not? | ☐ Yes ☐No |
|  | **The help button**  If yes, could you tell me something about your experience with this feature?  If not, why not? | ☐ Yes ☐No |
|  | **How easy or difficult was it to find a desired app in FindMyApps? Can you tell us why?**  Answer likely to fall under code B3 . | ☐Difficult ☐Somewhat difficult ☐Easy ☐Very easy |
|  | **What grade would you give the FindMyApps app in terms of general usability?**  Answer likely to fall under code B1 and/or B3. | 0 ..1 ..2 ..3 .. 4 .. 5 .. 6 .. 7.. 8 .. 9 .. 10 |
|  | **What grade would you give the FindMyApps app in terms of how much fun you had with it?**  Answer likely to fall under code B1(iii). | 0 ..1 ..2 ..3 .. 4 .. 5 .. 6 .. 7.. 8 .. 9 .. 10 |
|  | **Can you tell me something about your experiences with the apps you downloaded via the FindMyApps app?**  Possible follow-up questions:   - Could you easily use the apps which you/your partner downloaded? - Why/why not? - Which apps best met your interests?   Answer likely to fall under code B1(i-iii) and B4 . |  |
|  | **Did you also look anywhere else for new apps?**  If yes, why?  If yes, where did you look for apps?  Which apps did you find somewhere else which were fun and/or easy to use?  Answer likely to fall under code B1(i-iii), B3 and/or B4 . | ☐ Yes ☐ No  Why:  Where:  Write the names of apps mentioned: |
| **B2. Usefulness** | | |
|  | **In which ways did the tablet support you?**  Wait for answer. Having noted the answer, prompt with the following options:   - Self-management? - Meaningful activities? - Social contact?   Answer likely to fall under code B2 . | ☐Self-management ☐Meaningful activities ☐ Social contact ☐Other |
|  | **What grade would you give the tablet in terms of how useful it was?**  Answer likely to fall under code B2. | 0 ..1 ..2 ..3 .. 4 .. 5 .. 6 .. 7.. 8 .. 9 .. 10 |
| **B3. Learnability of tablet and FMA App** | | |
|  | **How did you experience learning to use the tablet? Can you tell me something about that?**  Answer likely to fall under code B3 . | ☐Difficult ☐Somewhat difficult ☐Easy ☐ Very easy |
|  | **How did you experience learning to use the FindMyApps app? Can you tell me something about that?**  Answer likely to fall under code B3 . | ☐Difficult ☐Somewhat difficult ☐Easy ☐Very easy |
|  | **Were you able to download apps yourself via the FindMyApps app? (with or without help from your partner)**  Answer likely to fall under code B3 . | ☐Yes ☐No |
|  | **Did the instruction films help you to learn to use the tablet and the FindMyApps app?**  Possible follow-up questions: If not, why not?  Answer likely to fall under code B3 . | ☐Yes ☐No |
|  | **Do you have suggestions to make learning to use the tablet easier?**  Answer likely to fall under code B3 . |  |
| **B4. ADOPTION of tablet and FMA app by PWD** | | |
|  | **To what extent is using the tablet part of your daily routine now?**  Follow-up:  **To what extent is using the FindMyApps app part of your daily routine now?**  Answer likely to fall under code B4 . |  |
| **C. Contextual factors** | | |
|  | **Before you started this research, what other technology or devices did you use regularly?**  Wait for answer before prompting with options (e.g. laptop, smartphone, etc.)  Answer likely to fall under code C | ☐ None/nothing ☐ Computer or laptop ☐ Smartphone (e.g. iPhone)  ☐ Other, namely: |
|  | **Before you started this research, did you already have experience using a tablet?**  Answer likely to fall under code C | ☐ Yes ☐ A little bit ☐ No |
| **GENERAL OBSERVATIONS** | | |
| / | How long did the interview last? |  |
| / | Other important contextual information? |  |

# SSI for the caregiver – Experimental Group – V1.0

Name of interviewer:

Code PwD and CG:

Date and time:

| 1. **Implementation** | | |
| --- | --- | --- |
|  | **Did the tablet work correctly during the research, as far as you’re concerned?**  Possible follow-up questions:   - Did you have problems turning the tablet on? - Did you have problems with the internet connection?   Answer likely to fall under code A1(i) . |  |
|  | **Did the FindMyApps app work correctly during the research, as far as you’re concerned?**  Possible follow-up questions:   - Did you have problems launching the FindMyApps app? - Did you have problems logging into the FindMyApps app?   Answer likely to fall under code A1(ii) . |  |
|  | **Can you tell me something about your experience with the training in errorless learning and the use of the tablet and FindMyApps app (which was given during the first appointment)?**  Possible follow-up questions:   - Could you follow the online training? - Did you find the training useful? - Did the level and topics meet your needs? - Did you succeed in applying what you learned?   Answer likely to fall under code A2 . |  |
|  | **Do you have suggestions for improving the training, for example, additional topics that you missed?**  Answer likely to fall under code A2 |  |
|  | **What was your experience of calling the ‘helpdesk’ for support?**  Answer likely to fall under code A1 | ☐ Not used |
|  | **How did you find it to explaining the functions of the tablet to your partner?**  Answer likely to fall under code B2 |  |
|  | **How did you find it to explaining the functions of the FindMyApps app to your partner?**  Answer likely to fall under code B2 |  |
|  | **Did the training help you to support your partner to learn to use the tablet and FindMyApps app?**  Wait for answer. Once answer noted, prompt with:   - Were you able to implement the ‘errorless learning’ method – as demonstrated during het training – to support your partner? - If not, why not?   Answer likely to fall under code B2 |  |
| **B. Mechanisms of Impact**  **B1. Usability** | | |
|  | **Which barriers did you encounter in using the tablet or FindMyApps app, or what made using the tablet and the FindMyApps app difficult?**  Answer likely to fall under code B1 |  |
|  | **What grade would you give the FindMyApps app in terms of general usability?**  Answer likely to fall under code B1 and/or B3. | 1. ..1 ..2 ..3 .. 4 .. 5 .. 6 .. 7.. 8 .. 9 .. 10 |
|  | **What grade would you give the FindMyApps app in terms of how much fun you had with it?**    Answer likely to fall under code B1(iii). | 1. ..1 ..2 ..3 .. 4 .. 5 .. 6 .. 7.. 8 .. 9 .. 10 |
| **B2. Usefulness** | | |
|  | **What grade would you give the tablet in terms of how useful it was?**  Answer likely to fall under code B2. | 1. ..1 ..2 ..3 .. 4 .. 5 .. 6 .. 7.. 8 .. 9 .. 10 |
| **B3. Learnability** | | |
|  | **Was your partner already able to download apps by themselves before this research?**  Answer likely to fall under code B3 | ☐Yes ☐ Sometimes ☐No ☐Not attempted |
|  | **Can your partner download apps by themselves now?**  Answer likely to fall under code B3 | ☐Yes ☐ Sometimes ☐No ☐Not attempted |
|  | **Can your partner use the tablet by themselves?**  Follow-up:  **Is your partner more or less proficient in using the tablet than before?**  Answer likely to fall under code B3 | ☐Yes ☐ Sometimes ☐No ☐Not attempted |
|  | **Can your partner use the FindMyApps app by themselves?**  Answer likely to fall under code B3 | ☐Yes ☐ Sometimes ☐No ☐Not attempted |
|  | **Heeft u suggesties om het gebruiken van en leren omgaan met de tablet en/of de FindMyApps app te vergemakkelijken?**  Answer likely to fall under code A, B1 and B3 |  |
| **B4. Adoption** | | |
|  | **To what extent is using the tablet part of your daily routine now?**  Follow-up:  **To what extent is using the FindMyApps app part of your daily routine now?**  Answer likely to fall under code B4 . |  |
| **C. Contextual Factors** | | |
|  | **Before you started this research, what other technology or devices did you use regularly?**  Wait for answer before prompting with options (e.g. laptop, smartphone, etc.)  Answer likely to fall under code C |  |
|  | **Before you started this research, did you already have experience using a tablet?**  Answer likely to fall under code C | ☐ Yes ☐ A little bit ☐ No |
|  | **Did material, social or financial factors influence your use of the tablet, the FindMyApps app or choice of apps?**   - Material factors include for example features of the home - Socal environment includes cohabiting with partner, for example   Answer likely to fall under code C |  |
| **GENERAL OBSERVATIONS** | | |
| / | How long did the interview last? |  |
| / | Other important contextual information? |  |

# Semi-structured interview questions for those implementing FindMyApps intervention

These questions can be posed to researchers who supported the implementation of the interventions during the FindMyApps project, by providing online tablet training and managing the helpdesk for participants (n = 3).

| **Was it possible to give training to all participants by video call?***  *Implementation* | Yes  No, because |
| --- | --- |
| **What did you think about video call as a medium for teaching tablet use?***  *Implementation, learnability* | Very appropriate  Appropriate  Not very appropriate  Completely inappropriate  Explanation: |
| **Do you think that the FindMyApps app functioned properly during the training sessions?**  *Implementation* | Always  Mostly  Sometimes  Rarely  Never  Explanation: |
| **Did you feel that a one-off training sessions was sufficiently effective, that participants could then use the tablet with support of their caregiver?**  **If yes, why?**  **If no, why not?**  *Learnability* | Always  Mostly  Sometimes  Rarely  Never  If so, this was due to ……  If not, this was due to ……. |
| **How did you find the experience of giving online training in use of the tablet and FindMyApps app to participants during the FindMyApps project?***  *Implementation, learnability* | Very stressful  A bit stressful  Relaxed  Very relaxed  Explanation: |
| **How easy did you fin dit to communicate with participants during the training?***  *Implementation, usability, learnability* | Very easy  Easy  Not very easy  Difficult  Explanation: |
| **Did you encounter any barriers or obstacles when giving the online training?**  *Implementation, context* | No  Yes, namely: |
| **What made giving the online training easier?**  *Implementation, context* |  |
| **How frequently were you contacted by participants (by phone or email) after the training, with questions about using the tablet or the FindMyApps app, or to inform you of problems they were having? (the helpdesk)**  *Learnability* | By … participants in total, called … times. |
| **During the follow-up calls (after 4 and 8 weeks) and if you were called or emailed via the helpdesk, what kind of questions or problems did people have?**  *Learnability* | Functioning of the tablet  Functioning of FindMyApps app  How to download apps  Other technical problems  Problems with motivation of the person with dementia  Insufficient time/other priorities  Problems with process of learning/teaching  Using tablet after end of study  Using FindMyApps after the study  Other, namely ………………………………. |
| **Do you have suggestions to improve the online training?*** |  |
| **Are there other experiences which you think may have influenced the training or the FindMyApps intervention?** |  |

*These questions were also put to participants regarding their experience of the online training via a survey at T1.
